# Efficient Heat Shock Response Affects Hyperthermia-Induced Radiosensitization in a Tumor Spheroid Control Probability Assay

**DOI:** 10.3390/cancers13133168

**Published:** 2021-06-25

**Authors:** Oleg Chen, Soňa Michlíková, Lisa Eckhardt, Marit Wondrak, Adriana M. De Mendoza, Mechthild Krause, Damian D. McLeod, Leoni A. Kunz-Schughart

**Affiliations:** 1 OncoRay—National Center for Radiation Research in Oncology, Faculty of Medicine and University Hospital Carl Gustav Carus, Technische Universität Dresden and Helmholtz-Zentrum Dresden – Rossendorf, 01307 Dresden, Germany; oleh.chen@gmail.com (O.C.); sona.michlikova@oncoray.de (S.M.); lisa.eckhardt@uniklinikum-dresden.de (L.E.); marit.wondrak@oncoray.de (M.W.); a.demendoza@javeriana.edu.co (A.M.D.M.); Mechthild.Krause@uniklinikum-dresden.de (M.K.); damian.mcleod@uni-oldenburg.de (D.D.M.); 2 Department of Cell Signaling, Institute of Cell Biology, National Academy of Sciences of Ukraine, 79005 Lviv, Ukraine; 3 Physics Department, Pontificia Universidad Javeriana, Bogotá 110231, Colombia; 4 German Cancer Consortium (DKTK), Partner Site Dresden and German Cancer Research Center (DKFZ), 69120 Heidelberg, Germany; 5 Department of Radiotherapy and Radiation Oncology, Faculty of Medicine and University Hospital Carl Gustav Carus, Technische Universität Dresden, 01307 Dresden, Germany; 6 Institute of Radiooncology—OncoRay, Helmholtz-Zentrum Dresden – Rossendorf, 01328 Dresden, Germany; 7 National Center for Tumor Diseases (NCT), Partner Site Dresden, 01307 Dresden, Germany; 8 School of Biomedical Sciences and Pharmacy, College of Health, Medicine and Wellbeing and Hunter Medical Research Institute, The University of Newcastle, Callaghan 2308, Australia; 9 School of Medicine and Health Sciences, Department of Cardiology, Carl von Ossietzky University of Oldenburg, 26129 Oldenburg, Germany

**Keywords:** hyperthermia, radiation therapy, proteotoxic stress, spheroids, heat shock proteins (Hsps), head and heck squamous cell carcinomas (HNSCC)

## Abstract

**Simple Summary:**

Resistance to therapy and subsequent relapse of the disease are common in patients with cancers in the head and neck region (HNSCC). Recent technological advancements have revitalized the concept of combining hyperthermia (HT) with radio(chemo)therapy for treating these patients. Heat inherently affects multiple cellular components and destroys protein structures, thereby influencing the DNA damage response. However, the plethora of adverse mechanisms in HT-induced radiosensitization is still not fully elucidated. We uniquely evaluated the radiosensitizing potential of HT in HNSCC cells using a sophisticated spheroid assay platform, which turned out as a powerful tool to compare different treatment modalities and gain new mechanistic insight. We show that HT disrupts vital cellular proteostasis and affects global stress response signaling. This triggers massive heat shock and proteotoxic stress responses contributing to the cancer cells’ protection against HT-induced radiosensitization. Selected molecules in this scenario may serve as new targets for combination with hyperthermia and radiotherapy.

**Abstract:**

Hyperthermia (HT) combined with irradiation is a well-known concept to improve the curative potential of radiotherapy. Technological progress has opened new avenues for thermoradiotherapy, even for recurrent head and neck squamous cell carcinomas (HNSCC). Preclinical evaluation of the curative radiosensitizing potential of various HT regimens remains ethically, economically, and technically challenging. One key objective of our study was to refine an advanced 3-D assay setup for HT + RT research and treatment testing. For the first time, HT-induced radiosensitization was systematically examined in two differently radioresponsive HNSCC spheroid models using the unique in vitro “curative” analytical endpoint of spheroid control probability. We further investigated the cellular stress response mechanisms underlying the HT-related radiosensitization process with the aim to unravel the impact of HT-induced proteotoxic stress on the overall radioresponse. HT disrupted the proteome’s thermal stability, causing severe proteotoxic stress. It strongly enhanced radiation efficacy and affected paramount survival and stress response signaling networks. Transcriptomics, q-PCR, and western blotting data revealed that HT + RT co-treatment critically triggers the heat shock response (HSR). Pre-treatment with chemical chaperones intensified the radiosensitizing effect, thereby suppressing HT-induced Hsp27 expression. Our data suggest that HT-induced radiosensitization is adversely affected by the proteotoxic stress response. Hence, we propose the inhibition of particular heat shock proteins as a targeting strategy to improve the outcome of combinatorial HT + RT.

## 1. Introduction

Recent technological advances in local hyperthermia (HT) delivery and monitoring have been critical for implementing thermoradiotherapy in standard clinical practice to treat various (mainly superficial) cancers, opening new attractive prospects in precision and personalized combinatorial treatments for deep-tissue tumors [1,2,3,4,5,6]. They also revitalized the attempts to combine HT with radio(chemo)therapy for treating head and neck squamous cell carcinoma (HNSCC) patients [7,8]. Different HT setups and treatment regimens may not induce comparable radiosensitizing responses—not even on the cellular level. Hence, ongoing efforts must systematically compare diverse treatment settings and options in HNSCC cell and tumor models, both in vitro and in vivo. Determining the most effective combination of HT and radiotherapy (RT) is clinically relevant. However, it proves particularly difficult because the treatment success depends on highly variable and flexible parameters such as treatment schedule and appropriate choice of radiation and thermal dose, the latter being defined by temperature and exposure time.

The most appropriate curative endpoints for radiotherapy outcome in vivo are the tumor control probability (TCP) and tumor control dose 50% (TCD_50_ = irradiation dose required to cure 50% of tumor-bearing animals) [9,10,11,12]. Such in vivo approaches are time-consuming, expensive, and ethically problematic. The main in vitro assays, on the other hand, have insufficient predictive power as they are still based on 2-D cell culture models, which neither resemble the 3-D morphology of cells and their nuclei nor do they adequately reflect the tumor microenvironment. Multicellular tumor spheroids (MCTS) mimic various therapeutically relevant pathophysiological characteristics of tumor microregions. These include 3-D cell-cell and cell-matrix interactions and in-vivo-like oxygen, nutrient, and proliferation gradients [13,14]. Furthermore, the 3-D cellular context and geometry are central regulators of gene expression, cellular signaling, and mechanotransduction [15,16,17]. Altogether, this makes tumor spheroids a valuable in vitro model with intermediate complexity to monitor the impact of HT on RT outcome. Nonetheless, it is not only the use of spheres or spheroids that counts, but it is also (and in particular) the analytical endpoints that can make sphere/spheroid platforms a valuable tool to systematically test HT + RT treatment parameters of interest.

Relevant pre-clinical and clinical endpoints to be reflected in MCTS assays are growth delay (non-curative) and long-term control probability (curative). From the less than one dozen studies using HT + RT in spheroid cultures [18,19,20,21,22,23,24,25,26,27,28], a few have indeed analyzed spheroid volume growth delay as a response to therapy [27,28]. However, therapeutic read-outs as pendants to TCP and TCD_50_ were not considered. The long-term monitoring analytical endpoints of spheroid control probability (SCP) and spheroid control dose 50% (SCD_50_ = irradiation dose to control 50% of the spheroids) have recently been established for combinatorial radiotherapy testing in spheroids, including HNSCC spheroid models [29,30,31], but have not yet been utilized in the context of HT + RT. Despite our ongoing attempts to improve the predictive value of these SCP assays, i.e., by including various stromal cell populations or by implementing clinically relevant fractionated irradiation regimes, we aim to provide a sophisticated tool that allows an improved pre-selection of the most promising HT + RT treatment settings from numerous options for further in vivo validation. The first step to achieve this is to adapt the basic read-out and demonstrate the SCP assay’s potential to quantify the thermal enhancement in radioresponse. Therefore, the present study uniquely evaluated the thermal dose-response relationship (37–46.5 °C) in HT-induced radiosensitization using multicellular HNSCC spheroid models and the proposed long-term analytical endpoints. The presented data based on >6000 spheroids and >120,000 images support the usefulness of the proposed assay to assess HT + RT settings.

Radiosensitization by HT in different cancer models can be attributed to diverse cellular effects [32,33]. HT alone disrupts thermal protein homeostasis (proteostasis) and induces heat shock (HSR) and unfolded protein (UPR) responses [34,35]. However, the consequences of HSR and UPR may essentially vary in different cell types, and for different thermal doses, duration of heat exposure, or when HT is combined with RT. Hence, they can have a strong protective function or switch to a cytotoxic response and cell death scenarios. The regulation of various chaperones integral to heat shock (HSR) and proteotoxic stress responses could thus foster or, more likely, adversely affect the radiosensitizing potential of HT. For example, Hsp27, Grp78, Hsp70, and Hsp90 might be upregulated in malignant cells [36], helping them adapt to stress conditions. The precise role of HSR and proteotoxic stress in HT-induced radiosensitization remains ambiguous. Therefore, we used our HNSCC spheroid model and assay to elucidate HSR and proteotoxic stress pathways upon HT and HT + RT via transcriptomics, qPCR/RT-PCR, and western blotting combined with the application of chemical chaperones. Our findings provide new fundamental insight into protective stress response mechanisms that promote survival in HNSCC spheroids upon HT + RT and support the use of the SCP assay as an analytical tool to pre-evaluate the potential of HT + RT combined with new (multi-)targeted therapies.

## 2. Materials and Methods

### 2.1. Cell Lines and Culture Conditions

Two human HNSCC cell lines were used in this study: SAS cells obtained from the HSRRB/JCRB (Osaka, Japan) and FaDu cells, a subline of the FaDu-ATCC HTB-43 [37]. Both cell lines were routinely tested free of mycoplasms using a PCR Mycoplasma Kit (AppliChem, Darmstadt, Germany), and the genetic profile was verified before use via microsatellite analyses using multiplex PCR kits as detailed previously [38]. Cultures were routinely grown from validated frozen stocks for ≥2 to ≤20 passages (<120 cumulative population doublings).

The cells were cultured as monolayers in standard Dulbecco’s Modified Eagle Medium (DMEM) with L-glutamine, D-glucose (1 g/L) and 25 mM HEPES supplemented with 10% heat-inactivated fetal calf serum (FCS) and 1% penicillin/streptomycin (10,000 U/mL/10 mg/mL). Cells were kept at 37 °C in a humidified atmosphere with 8% CO_2_. Exponentially growing cultures were enzymatically dissociated using 0.05% trypsin/0.02% EDTA in phosphate-buffered saline (PBS) to obtain single-cell suspensions for passaging and spheroid initiation. All culture media, supplements, solutions, and buffers were purchased from PAN-Biotech (Aidenbach, Germany). A CASY^®^ TTC analyzer (Roche Innovatis, Reutlingen, Germany) was used to monitor cell culture quality and assess cell numbers and volumes in single-cell suspensions for further processing.

### 2.2. Spheroid Routine Culturing and Monitoring

Spheroids were cultured in liquid overlay as described earlier [29]. In brief, 800–1000 SAS or 1800–2000 FaDu cells derived from exponentially growing monolayer cultures were seeded in 200 µL of regular DMEM per well in 96-well plates coated with 1.5% agarose (Sigma-Aldrich Chemie, Taufkirchen, Germany). All experiments were performed after 4 days of incubation when spheroids of both cell lines reached a mean diameter ~400 µm. A Biomek 4000 Automated Liquid Handler (Beckman Coulter, Brea, CA, USA) was used to feed spheroids every 48–72 h by exchanging 50% of the supernatant with standard DMEM. Spheroid integrity and morphology were routinely monitored, and growth kinetics was assessed by semi-automated measurements of spheroid diameters (and volumes) either from phase-contrast images taken with an automated Axiovert 200M microscope (Carl Zeiss, Oberkochen, Germany) as detailed in [29,31] or from bright-field images acquired with a Cytation5 Imaging System (BioTek Instruments, Winooski, VT, USA). The latter was set up for 96-well agarose-coated plates to produce a Z-stack projection from six images taken per spheroid (4× objective; bottom image setting: −30 µm from the vertical center of the well, step size: 150 µm).

### 2.3. Hyperthermia and Irradiation Treatment

Prior to all treatments, the supernatant in the 96-well plates was reduced to 100 µL per spheroids and well, respectively. Plates were then sealed with ThermalSeal^®^ thermoresistant sealing film (Excel Scientific, Victorville, CA, USA) and placed in a pre-heated temperature-controlled PST-60HL-4 Plate Thermo-Shaker (BioSan, Riga, Latvia) for a time-defined exposure to HT. Control spheroids were incubated in parallel in sealed plates at 37 °C. The heating profiles in the wells of agarose-coated 96-well plates were recorded for different temperature settings via a TC-08 8 channel thermocouple data logger (Pico Technology, Cambridgeshire, UK) combined with type T thermocouples (RS Components, Corby, UK) and analyzed before using the system for standardized HT treatment (Figure S1a). The actual temperature was 0.5 °C higher than the setting of the device. 95% of the target temperature was reached within 10 min. The treatment times stated in this study comprise the entire heating period, starting with the placement of the plates in the device.

For RT treatment, spheroids were irradiated in the 96-well plates at room temperature using single doses of 0–25 Gy (200 kV X-rays; 0.5-mm Cu filter; YxlonY.TU 320; Yxlon International, Hamburg, Germany). In the combination treatment settings, irradiation was applied immediately after HT exposure (within ~1 min). Upon completion of the treatment, 100 µL of fresh medium were added to each well, and the plates were transferred back to standard culture conditions. The spheroids were then routinely fed as described before and monitored for up to 60 days or collected at defined time points (0.5–24 h after HT or HT/RT) for further processing and molecular analyses.

### 2.4. Exposure to Chemical Chaperones

Two different chemical chaperones were applied in this study—tauroursodeoxycholic acid (TUDCA, Merck, Darmstadt, Germany) and sodium phenylbutyrate (4-PBA, Cayman Chemical, Ann Arbor, MI, USA). TUDCA and 4-PBA were always freshly dissolved in deionized H_2_O at 8 mM and 100 mM, respectively, and further diluted 1:10 in supplemented DMEM directly before use. For spheroid exposure, the supernatant was reduced to 100 µL per well and re-supplemented by 100 µL of the chaperone solution to reach final concentrations for the treatment of 0.4 mM for TUDCA and 5 mM for 4-PBA. DMEM with an equivalent H_2_O content was used as vehicle control. After 1 h of incubation, 50% of the chaperone-containing media were removed, plates were sealed, and the treatment procedure continued as described above. In the long-term experiments, exposure to the chaperones was terminated after 24 h by careful washing and transferring the spheroids into fresh agarose-coated 96-well plates using the automated pipetting system equipped with wide-bore tips.

### 2.5. Spheroid Volume Growth Delay and Spheroid Control Probability (SCP) Assays

All spheroids were imaged individually directly before treatment and every 48–72 h thereafter until they reached ~900 µm in diameter. Spheroid sizes and subsequent volumes were determined from the images as described [29,38]. Relative growth delay was calculated as a time interval of each spheroid in a treatment arm to reach 5-times its initial volume before the onset of treatment (5 × V_0_) divided by the average time required by an untreated spheroid control population. For this purpose, we identified the monitoring time points closest to the individual 5 × V_0_ and calculated the individual times to reach 5 × V_0_ via regression analyses through the logarithm of the corresponding spheroid volumes. The Mann-Whitney U test was used for statistical comparison.

The spheroid control probability assay was basically carried out as previously highlighted [30,31]. Spheroid integrity and size was monitored routinely over a period of up to 60 days post-treatment. Spheroids which did not recover growth within the observation time and enlarged over at least three consecutive time points were declared as controlled (Figure S1b). The proportions of controlled spheroids as function of time after treatment are illustrated as Kaplan-Meier curves and were statistically compared using the Log-rank (Mantel-Cox) test. The SCP refers to the proportion of spheroids that lost regrowth capacity in a particular treatment arm. SCP values were recorded as function of the irradiation dose. An in-house Python-based program was used to generate spheroid dose-response curves by fitting a logistic regression dose-response model (1) according to the tumor control probability in vivo assay [39]: (*D*: irradiation dose; *a*,*b*: variables):

(1) $$\mathrm{SCP}=\frac{1}{1+e^{\left(-a-bD\right)}}$$

Every SCP dose-response curve comprises eleven irradiation treatment arms (0, 2.5, 5, 7.5, 10, 12.5, 15, 17.5, 20, 22.5, and 25 Gy) and derived from the monitoring of roughly 600 spheroids which refers to a set of ~15,000 images. Subsequently, the spheroid control dose 50 (SCD_50_) was calculated as the dose leading to an SCP of 50% (= loss of regrowth capacity in 50% of the spheroid population). Bootstrapping with 4000 samples was carried out to determine 95% confidence intervals and estimate statistical significance. The thermal enhancement ratio (TER) was calculated as the ratio of the computed SCD_50_ values in spheroids treated with RT alone vs. HT + RT combination treatment.

All spheroid volume growth and SCP data documented in our study originate from N = 2 independent experiments for each cell model, with a total of ∑n ≥ 56 spheroids per treatment arm (up to 5 HT × 11 RT doses = 55 treatment arms; *n* ≥ 26 spheroids per treatment arm and experiment). All statistical analyses were performed with pooled data sets.

### 2.6. Reverse Transcription (RT-) and Real-Time Quantitative-(q-)PCR Analyses

Total cellular RNA was isolated from SAS and FaDu spheroids using the RNeasy Mini kit (QIAGEN, Hilden, Germany) according to the manufacturer’s protocol. RNA concentrations and quality were verified with a Nanodrop One spectrophotometer (Thermo Fisher Scientific, Waltham, MA, USA). 0.5 µg of total RNA was used for cDNA synthesis with the Verso cDNA Synthesis Kit (Thermo Fisher Scientific); cDNA was diluted (1:10), and a one-step PCR reaction was performed using the GoTaq qPCR Master Mix (Promega, Madison, WI, USA) according to the manufacturer’s instructions. Data were collected and analyzed with the Applied Biosystems StepOnePlus Real-Time PCR System and the v.2.2.2 StepOne Software (Life Technologies, Applied Biosystems, Darmstadt, Germany). The relative gene expression of control versus treated cells was assessed by the comparative threshold cycle (ΔΔCt) method with *ACTB* gene expression serving as reference.

Classical PCR was carried out in an MJ Research PTC-200 Thermal Cycler (Bio-Rad, Hercules, CA, USA) using the GoTaq Flexi DNA Polymerase Kit (Promega) with specific primers for *XBP1* (spliced and unspliced form) or selected Hsps. Conditions for the PCRs were as follows: initial denaturation at 95 °C for 5 min, denaturation in cycles at 95 °C for 30 s, annealing at 55 °C for 30 s, synthesis at 72 °C for 1 min, and a final extension step at 72 °C for 7 min. PCR products and GeneRuler 100 bp Plus DNA Ladder (Thermo Fisher Scientific) were separated by 2.0% or 4.0% agarose gel electrophoresis, visualized via RedSafe (iNtRON Biotechnology, Seongnam-Si, Korea) dye staining, and documented using the GeneGenius Gel Imaging System (Syngene, Cambridge, UK). Table S1 lists all primer pairs with product sizes. All values from the mRNA expression analyses are presented as mean (+SD) from three independent experiments, each with ≥2 technical replicates (N = 3, *n* ≥ 2). Treatment arms were compared to the untreated controls using a paired Student’s *t*-test.

### 2.7. Western Blotting

Western blots were performed using whole-cell protein extracts from 3-D cultures as described previously [40]. Protein content was determined with the Pierce BCA Protein Assay Kit (Thermo Fisher Scientific) as described by the manufacturer. Samples were separated by gradient 4–20% SDS-PAGE Precast protein gels (Bio-Rad) and transferred onto nitrocellulose membranes (Whatman, GE Healthcare, Dassel, Germany). Diverse primary antibodies (see Table S2) were applied according to the manufacturers’ instructions to label specific non-phosphorylated and phosphorylated proteins of interest. Horseradish peroxidase-conjugated goat anti-mouse or anti-rabbit IgG secondary antibodies (Cell Signalling Technology, Leiden, Netherlands) were applied for signal detection. The immunoreactive bands were visualized with a chemiluminescence detection kit (Santa Cruz Biotechnology, Dallas, TX, USA) and documented using a ChemiDoc Imaging System (Bio-Rad). GAPDH protein levels were used as loading controls. All experiments were performed in triplicate (N = 3); reproducible observations are illustrated by representative Western blot images.

### 2.8. Transcriptomics

For RNA-Seq, total RNA was isolated from FaDu and SAS spheroids as described above in PCR analyses. RNA samples from N = 3 independent experiments in both spheroid models were quantified using a Qubit 2.0 Fluorometer (Life Technologies, Darmstadt, Germany). The RNA integrity was verified with an Agilent Fragment Analyzer (Agilent Technologies, Santa Clara, CA, USA). RNA-Seq libraries were prepared and sequenced by GENEWIZ (Leipzig, Germany) with an Illumina NovaSeq™ 6000 system. Raw sequence data (.bcl files) were converted into FASTQ files and de-multiplexed via the Illumina bcl2fastq 2.19 Software. Differential gene expression was analyzed with the DESeq2 normalization approach. The Wald test was used to generate adjusted *p*-values and log2 fold changes in expression for HT/RT treatments of interest relative to control spheroids. Genes with an adjusted *p*-value < 0.05 and absolute log2 fold change >1 were classified as differentially expressed. These genes were clustered by their gene ontology (GO), and the enrichment of GO terms was tested for significance using the Fisher’s exact test (GeneSCF v1.1-p2). The accession number for the RNA sequencing data reported in this paper is NCBI GEO: GSE150922.

## 3. Results

### 3.1. HT Induces Minimal Spheroid Growth Delay at Clinically Relevant Temperatures

**Figure 1:**
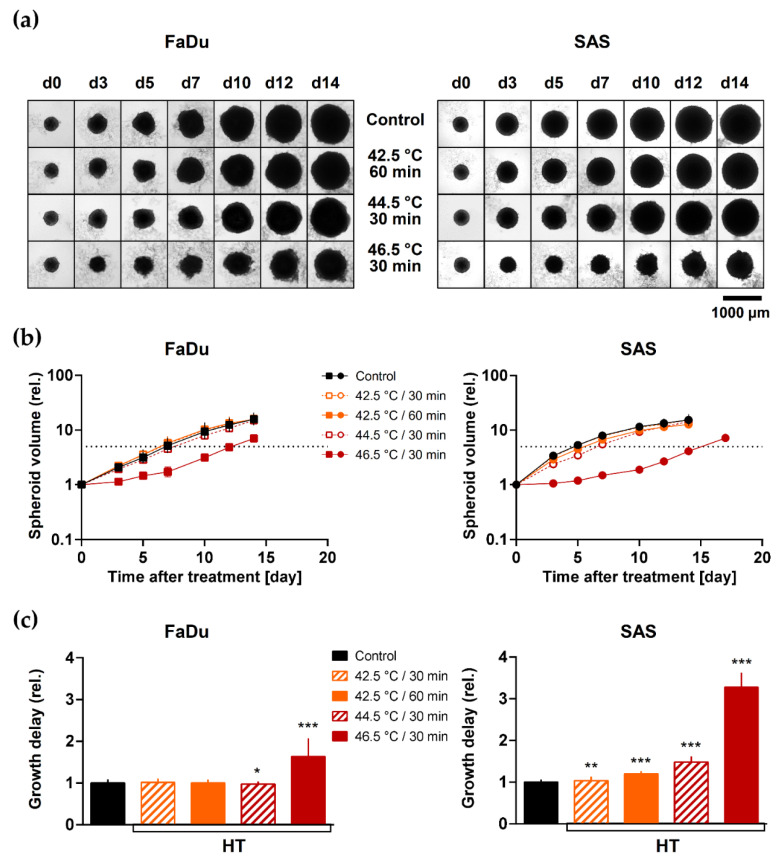
HT induces thermal dose-dependent HNSCC spheroid volume growth delay: (**a**) Representative image series of FaDu and SAS spheroids upon exposure to HT demonstrate the impact of various thermal doses on spheroid growth. (**b**) Effect of different hyperthermia treatments and thermal doses, respectively, on FaDu and SAS spheroid volume growth up to 14 day of post-treatment. Data show means (±SD) of ∑n ≥ 56 spheroids per treatment arm from N = 2 independent experiments. The dotted line indicates the relative value for 5 × V_0_. (**c**) Mean relative spheroid growth delay (±SD) induced by different thermal doses calculated from the data presented in (**b**) as the extended time to reach 5× pre-treatment spheroid volume (5 × V_0_) is shown to enable the comparison of the different spheroid types; treatment arms were normalized to the respective untreated controls. The Mann-Whitney U test was applied to estimate statistical significance of the relative growth delay (treated versus control spheroids) based on the pooled data; * *p* < 0.05; ** *p* < 0.01; *** *p* < 0.001.

First, we analyzed volume growth delay induced by various HT treatments alone. Figure 1a shown the morphology of representative FaDu and SAS spheroids as function of time after exposure, and Figure 1b documents the mean volume growth kinetics of multiple spheroids treated with different HT doses. Growth delay of each individual spheroid was calculated as the time required to reach 5× its initial volume before treatment (V_0_) relative to the time needed by untreated control spheroids. The analytical data reveal that HT at 42.5 °C for 30 and 60 min had little or no effect on spheroid volume growth (Figure 1c, Table S3). Only more severe HT at higher temperatures (46.5 °C) caused a substantial growth delay in both spheroid types. The HT effect on volume growth was in general stronger in SAS than FaDu spheroids.

### 3.2. HT Induces Sensitization of HNSCC Spheroids to Single Dose Irradiation

**Figure 2:**
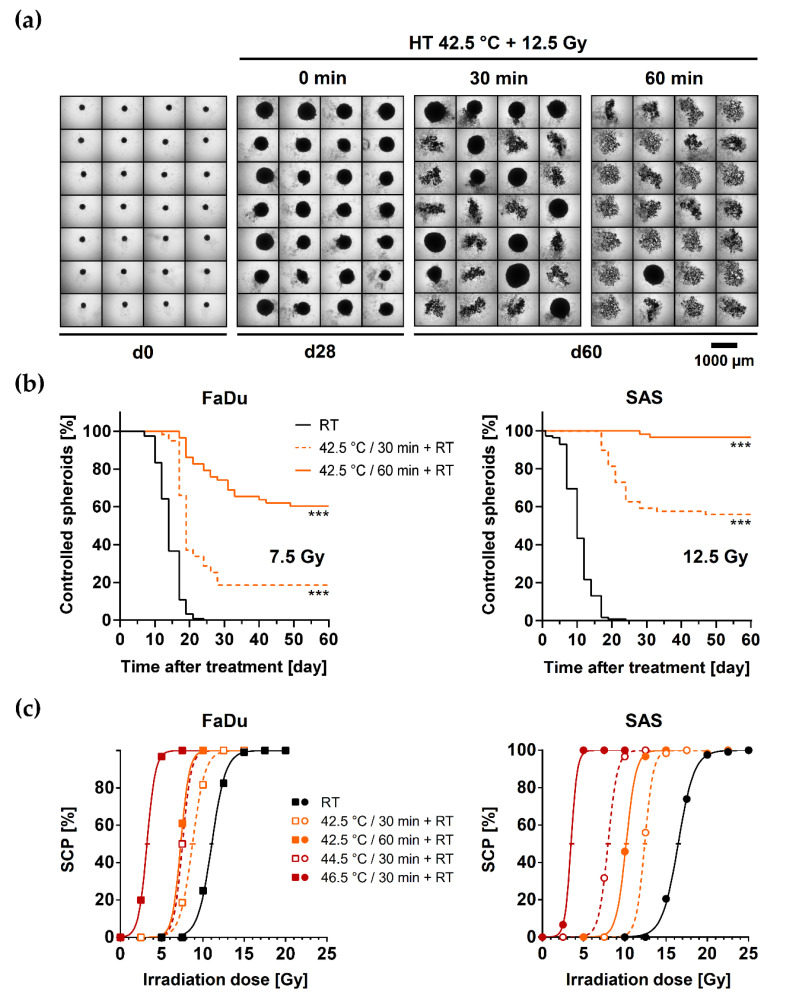
HT efficiently sensitizes both HNSCC spheroid types to single dose irradiation: (**a**) Representative images of 28 SAS spheroids exposed for 30 min or 60 min to 42.5 °C followed by 12.5 Gy single dose irradiation illustrate the radiosensitizing effect of different doses of HT; spheroids before treatment and without (0 min) HT pre-exposure are documented for comparison. (**b**) Proportions of controlled (non-regrown) FaDu and SAS spheroids documented as function of time post-treatment when exposed for 0, 30, or 60 min to 42.5 °C HT before single dose irradiation (FaDu—7.5 Gy; SAS—12.5 Gy); N = 2; ∑n ≥ 56 spheroids per condition; *** *p* < 0.001 as assessed with the Log-rank (Mantel-Cox) test for survival curves based on the pooled data. (**c**) Spheroid control dose response curves after HT pre-exposure and 0–25 Gy single dose irradiation; the proportion of spheroids that lost regrowth capacity (spheroid control probability, SCP) is recorded as a function of the irradiation dose. Every data point in each HT treatment arm represents the SCP of ∑n ≥ 56 individual spheroids from N = 2 independent experiments monitored up to 60 days post-treatment (≥560–620 spheroids per SCP curve). Horizontal bars present the 95% confidence interval of the SCD_50_ (spheroid control dose 50%).

We next performed long-term spheroid control probability (SCP) assays to address the putative ‘curative’ radiosensitizing potential of different thermal doses. FaDu and SAS spheroids can growth arrest, shrink or disintegrate after irradiation. Subsequently, they show a radiation dose-dependent capacity to regrow after treatment (Figure S1b). The potential to recover is reflected by the proportion of ‘controlled’ spheroids in a spheroid population, i.e., those that cannot regrow over 60 days post-treatment. The corresponding SCP allows the generation of SCP dose-response curves and the calculation of the SCD_50_ as an in vitro pendent to the radiotherapeutically relevant pre-clinical curative endpoint of TCP_50_ in vivo. The SCD_50_ values for FaDu and SAS spheroids after irradiation alone were 11.1 Gy and 16.5 Gy, respectively. The difference reflects the SAS model’s higher intrinsic radioresistance, consistent with previous reports on both spheroid and mouse xenograft experiments [11,12,30,38]. Figure 2a illustrates the increasing loss in growth recovery in an SAS spheroid population when RT is applied after 30 or 60 min of HT at 42.5 °C. Figure 2b depicts the HT-dependent re-growth capacity of FaDu and SAS spheroids as a function of time post-treatment for one irradiation dose of interest. Each time course in Figure 2b derives from the monitoring of ≥56 spheroids and refers to one data point in Figure 2c, which documents the irradiation-dose dependent SCP values. The combination of various HT doses with irradiation resulted in distinct left shifts of the SCP curves and decremental SCD_50_ values (Figure 2c and Table 1). The decrease in SCD_50_ was spheroid-type specific and depended on the thermal dose. Thermal enhancement ratios (TER) were calculated to quantitatively assess the radiosensitizing efficacy of different thermal doses in both spheroid types (Table 1). The TER is defined as the ratio of the SCD_50_ values upon RT mono- and HT + RT co-treatment. The results reveal the efficient, thermal dose-dependent HT-induced radiosensitization in both FaDu and SAS spheroids. However, the effect was more pronounced in the radioresistant SAS model, which proved to be more thermosensitive. Notably, both spheroid types were substantially radiosensitized (TER = 1.3–1.6) by HT doses that per se did not affect the spheroid volume growth kinetics (Table S3/Figure 2c *cf.* Figure 1b).

### 3.3. Global Changes in Gene Expression Profiles of HNSCC Spheroids upon HT + RT

To identify pathways of interest related to treatment response, we performed RNA-Seq analysis on FaDu and SAS spheroids exposed to a clinically relevant HT dose of 42.5 °C for 60 min followed by single-dose irradiation. The applied irradiation dose should *per se* result in comparable radioresponse and be close to the SCD_50_ for best evaluation; therefore, we irradiated FaDu spheroids with 7 Gy and SAS spheroids with 10 Gy.

**Figure 3:**
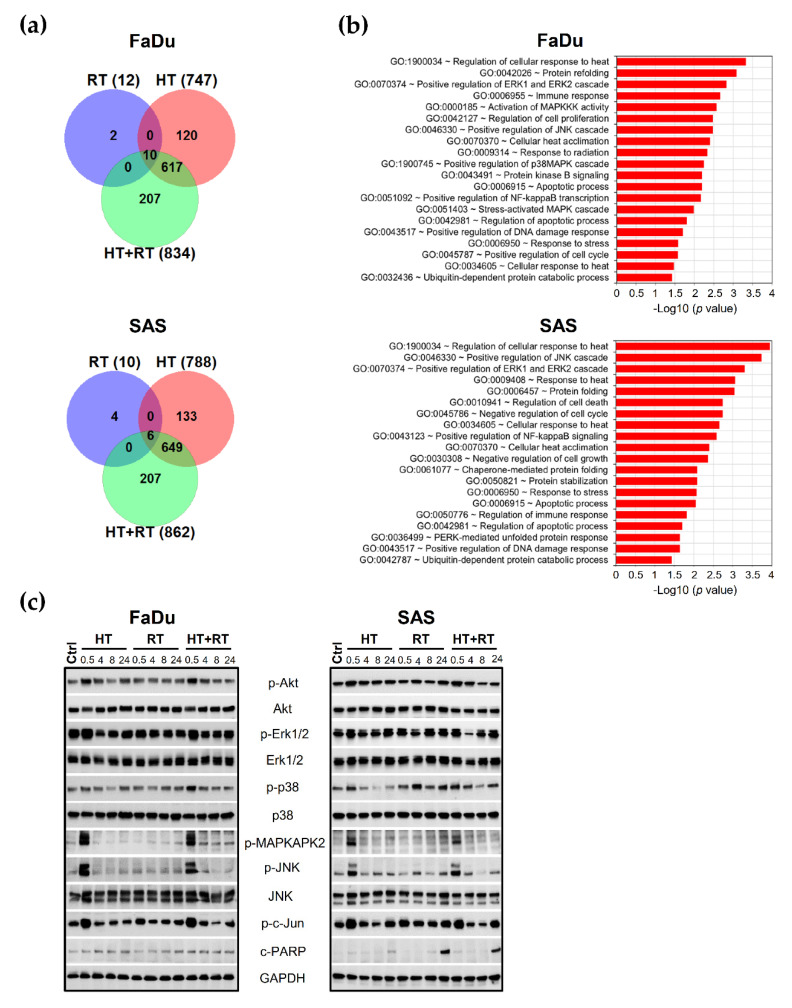
Combination of HT with RT affects the main signaling networks and stress response pathways in HNSCC spheroids: (**a**) Venn diagrams illustrating the number of differentially expressed genes from the whole genome for RT, HT, and HT + RT-treated FaDu and SAS spheroids in triplicate (0.5 h after treatment). Genes were selected by DESeq2 with at least 2-fold changes in expression relative to the appropriate control spheroids (adjusted *p* ≤ 0.05). (**b**) The top 20 selected signaling pathways and processes that are significantly (2-fold change or more, adjusted *p* ≤ 0.05) over-represented in FaDu and SAS spheroids 0.5 h after exposure to HT + RT (42.5 °C/60 min; 7 Gy for FaDu and 10 Gy for SAS) when compared to controls using the GO enrichment analysis. The *X*-axis presents the corresponding adjusted *p* values according to Fischer exact’s test (in negative log10 scale). (**c**) Representative Western blot data sets showing the expression/activation of proteins of interest from the MAPK and PKB/Akt signaling pathways in the two HNSCC spheroid types upon treatment. GAPDH was used as loading control. Spheroid treatment conditions: Ctrl—control; RT—single dose irradiation (7 Gy for FaDu and 10 Gy for SAS); HT—hyperthermia (42.5 ˚C/60 min); HT + RT—hyperthermia and single dose irradiation according to mono-treatments.

Gene signatures of control, RT, HT, and HT + RT treated spheroids were directly compared (0.5 h post-treatment). The data revealed that RT alone altered the expression of only 12 and 10 genes while 747 and 788 genes were specifically modulated (2-fold changes; *p* ≤ 0.05) by HT in FaDu and SAS spheroids, respectively (see Venn diagram in Figure 3a, Table S5). The combination of HT with RT did not counteract HT-triggered signaling and resulted in a higher number of altered genes than each treatment alone (834 genes in FaDu and 862 genes in SAS spheroids). However, the gene alterations included here that were modified exclusively by HT + RT (207 genes in each spheroid model) represent quite diverse genes and transcripts with minor or unknown functions, e.g., ~60–70 antisense transcripts, ~20 long non-coding RNAs (lncRNA), some adaptor and scaffold proteins or random genes, etc. These cannot yet be interpreted in the context of treatment outcome, also because there was only minor overlap in the genes uniquely altered upon HT + RT in FaDu and SAS spheroids (5/207).

The main pathways and networks modulated by the HT + RT combination (0.5 h after treatment) were identified using Gene Ontology (GO) enrichment analysis based on all 834 (FaDu) and 862 (SAS) significantly regulated genes compared to untreated control spheroids. Figure 3b highlights the top enriched pathways in the two spheroid types after treatment with HT + RT. It documents the up-regulation of selected canonical GO terms related to the cellular response to heat and stress, protein refolding and stabilization, heat acclimatization, as well as protein degradation signaling. Other GO terms of interest related to survival, adaptation, cell proliferation, cell cycle regulation, and apoptosis processes were also among the most represented pathways, namely stress- responsive MAPK and protein kinase B/Akt signaling (Figure 3b, Table S6). Furthermore, genes associated with DNA damage and immune responses were highly enriched. Notably, the majority of significantly modulated genes under HT and HT + RT are overlapping (617 and 655 genes for FaDu and SAS spheroids, respectively). Accordingly, there was no considerable difference in GO terms under HT alone compared to HT + RT treatment in both spheroid models (Table S6) underlining the prominent role of HT in gene regulation and suggesting that basically HT triggers the most relevant changes in the transcriptome of the HNSCC cells when exposed to HT + RT.

### 3.4. HT Affects Main Survival and Stress Response Signaling in HNSCC Spheroids

Based on the RNA-seq data, we next examined how HT alone and combinatorial HT + RT alters the activation status of the paramount survival and stress response pathways on the protein level (0.5–24 h after treatment). In both, FaDu and SAS spheroids, a rapid and transient increase (within 30 min) in the activated Akt protein (phosphorylated at Ser473; extremely augments Akt activity) occurred after HT with the total level of Akt protein been unaffected (Figure 3c). Furthermore, HT alone and in combination with RT transiently increased the activation and phosphorylation of all three branches of the MAPK cascade: extracellular regulated protein kinase 1/2 (Erk1/2), p38 kinase, and c-Jun N-terminal kinases (JNK) (Figure 3c). Also, the intermediate MAPKAPK2 kinase was found to be activated; MAPKAPK2 is a downstream target of p38 kinase and mediates phosphorylation of Hsp27 in response to stress [41]. More importantly, HT alone and in combination with RT dramatically enhances the active form (phosphorylated at Ser73) of the c-Jun transcription factor (Figure 3c), a well-known target of JNK kinase, which is influenced by various stress stimuli and contributes to apoptosis in many cell types [42,43].

Notably, SAS spheroids that are more heat-sensitive but more radioresistant showed a more pronounced activation of p38 stress kinase and c-Jun within 24 h of RT treatment than FaDu spheroids. Besides, an accumulation of the caspase substrate poly(ADP-ribose) polymerase protein 1 cleaved form (cPARP) protein, as the classical apoptotic marker, was detected 24 h after the RT and HT + RT treatments exclusively in SAS spheroids (Figure 3c). There were no changes in the levels of p-Akt, p-Erk1/2, and p-JNK in RT-treated spheroids up to 24 h post-treatment. In summary, our results demonstrate that mild HT alone or in combination with RT triggers a stress-activated MAP kinase cascade, including p38, JNK, and c-Jun transcription factor activation.

### 3.5. HT Triggers Heat Shock Response (HSR) and Proteotoxic Stress in HNSCC Spheroids

**Figure 4:**
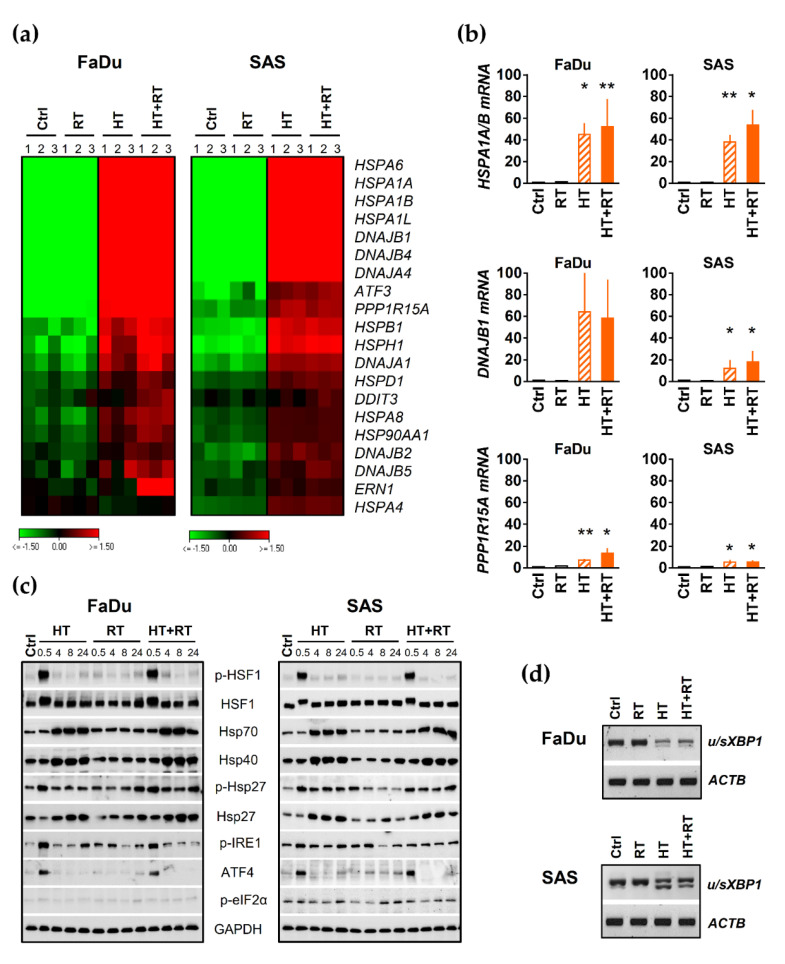
HT triggers heat shock response and proteotoxic stress in HNSCC spheroids: (**a**) Heat map of differentially expressed HSR and UPR genes in FaDu and SAS spheroids within 0.5 h of treatments profiled by RNA-Seq analysis in triplicate. Genes were selected by DESeq2 with at least 2-fold changes in expression under HT + RT treatment as highlighted in Figure 3, relative to appropriate control spheroids (adjusted *p* ≤ 0.05). The color-coded data represent log2 values reflecting down-regulation in green and up-regulation in red. (**b**) q-PCR analysis of three selected genes identified as upregulated by HT and HT + RT treatments in FaDu and SAS spheroids in our RNA-Seq analysis. Data were normalized to *ACTB* gene expression and are shown as means (±SD) of N = 3 independent experiments; * *p* ≤ 0.05; ** *p* ≤ 0.01. (**c**) Western blot data sets showing the expression/activation of HSR and UPR proteins of interest in FaDu and SAS spheroids 0.5–24 h after exposure to HT and/or irradiation; GAPDH was used as loading control. (**d**) Representative PCR analysis documenting the splicing of *XBP1* mRNA in FaDu and SAS spheroids 0.5 h after HT, RT and HT + RT treatments; the *ACTB* gene expression served as reference. Spheroid treatment conditions: Ctrl—control; RT—single dose irradiation (7 Gy for FaDu and 10 Gy for SAS); HT—hyperthermia (42.5 °C/60 min); HT + RT—hyperthermia and single dose irradiation according to mono-treatments.

We proceeded by evaluating the impact of HT alone and in combination with RT on the expression of several chaperones and ER stress markers as key components of the HSR and UPR signaling pathways. As before, transcription levels were mainly analyzed 0.5 h after treatment, and protein expression was monitored over 0.5–24 h post-treatment. The transcriptomic data revealed a significant induction of HSR signature genes in both HNSCC spheroid types upon HT and HT + RT, i.e., the expression of some genes of the Hsp70 and Hsp40 families was substantially enhanced, such as *HSPA6*, *HSPA1A*, *HSPA1B*, *HSPA1L*, as well as *DNAJA4*, *DNAJB1*, and *DNAJB4* (Figure 4a). qPCR and Western blot analyses of selected chaperones verified the observation (Figure 4b,c). Both total and active phosphorylated form (Ser326) of HSF1 (key transcription factor of HSR) were dramatically enhanced 0.5 h after HT or HT/RT treatment (Figure 4c). The effect was transient and reverted within four hours in both tested models. However, it is worth emphasizing that RT alone did not affect the expression of the same HSR genes/molecules in the two spheroid models. It also neither intensified nor diminished the HT-triggered Hsp upregulation or activation (Figure 4b,c). Taken together, HT with and without RT caused a significant upregulation/activation (phosphorylation) of key chaperones indicating substantial deregulation of proteostasis.

The transcriptomic data further implied that HT and HT + RT but not RT mono-treatment results in the activation of UPR signaling and significant induction of *PPP1R15A* (GADD34), *ATF3*, *DDIT3* (CHOP), and *ERN1* (IRE1α) signature genes in both FaDu and SAS spheroids (Figure 4a). Indeed, HT alone and in combination with RT caused a reproducible transient IRE1α activation (phosphorylation of Ser724) (Figure 4c). Activated IRE1α supposedly cleaves its pre-mRNA *XBP1* substrate to produce active spliced sXBP1 protein [44]. Accordingly, we detected sXBP in the two HNSCC models upon HT mono- and combinatorial treatment (Figure 4d, lower bands). Like IRE1α activation, HT/HT + RT induced a rapid but transient (only up to 4 h post-treatment) induction of ATF4 protein in both spheroid types.

Next, we monitored protein translation suppression by analyzing the expression level of GADD34, a regulatory subunit of eIF2α phosphatase PP1. The corresponding mRNA (*PPP1R15A*) was upregulated in HT/HT + RT-treated FaDu (more pronounced) and SAS spheroid cultures compared to untreated controls (Figure 4a,b). These results confirm that HT disrupts the proteome’s thermal stability, causing proteotoxic stress in both SAS and FaDu spheroids. RT-PCR analyses of selected genes in the two spheroid models over a 24 h time period upon exposure to increasing thermal doses support this conclusion, as HT at higher temperatures induced more severe and prolonged proteome damages and UPR-signaling (Figure S2).

### 3.6. HT-Induced Radiosensitization Is Adversely Affected by the Proteotoxic Stress Response

**Figure 5:**
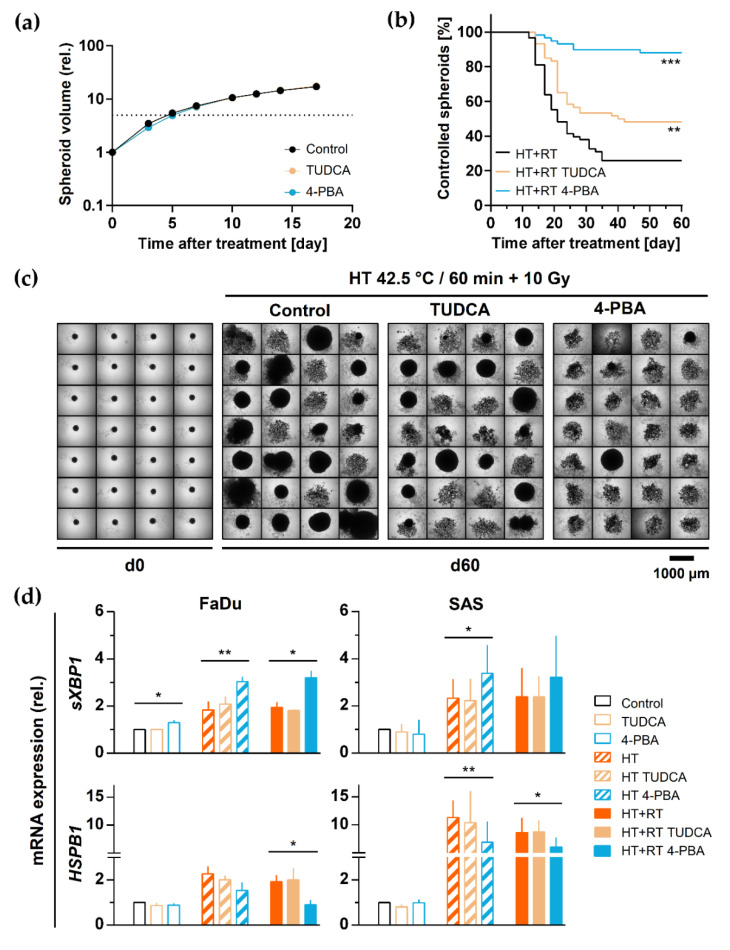
Induction of proteotoxic stress plays a protective role in HNSCC spheroids against HT-induced radiosensitization: (**a**) 24 h treatment with the chemical chaperones TUDCA (0.4 mM) and 4-PBA (5 mM) alone does not affect SAS spheroid volume growth kinetics. Data points show mean spheroid volumes of ∑n ≥ 56 spheroids per treatment arm from N = 2 independent experiments (±SD). (**b**) Proportion of controlled SAS spheroids irradiated with 10 Gy directly after exposure to 42.5 °C for 60 min in the absence or presence of TUDCA (0.4 mM) or 4-PBA (5 mM); the time courses represent the monitoring of ∑n ≥ 56 spheroids per treatment arm from N = 2 independent experiments over a period of 60 days post-treatment. ** *p* < 0.01 and *** *p* < 0.001 as assessed with the Log-rank (Mantel-Cox) test for survival curves based on the pooled data. (**c**) Images of 28 representative SAS spheroids before and at day 60 after treatment with HT + RT with or without TUDCA or 4-PBA according to (**b**). (**d**) qPCR analysis of *sXBP1* and *HSPB1* gene expression 0.5 h after HT (42 °C, 60 min) with or without RT (7 Gy—FaDu, 10 Gy—SAS) in the absence and presence of TUDCA or 4-PBA according to (b,c); data were normalized to *ACTB* gene expression and are shown as means (+SD) of N = 3 independent experiments; * *p* < 0.05; ** *p* < 0.01.

To functionally assess the role of proteotoxic stress and HSR response in HT-induced radiosensitization, we analyzed the impact of the two differently acting chemical chaperones TUDCA and 4-PBA. SAS spheroids were pre-exposed to the drugs (0.4 mM TUDCA; 5 mM PBA) 1 h before and during HT + RT treatment to stabilize the misfolded proteins artificially, suppress the unfolded protein aggregation, and alter the proteotoxic stress response. None of the chemicals alone was toxic at the defined concentration or substantially affected SAS spheroids’ regrowth potential after irradiation alone (Figure 5a). Interestingly, HT-induced radiosensitization in SAS spheroids was significantly improved by 4-PBA and partially by TUDCA. The proportion of controlled spheroids increased from 26% in the HT + RT treatment arm to 48% and 88%, respectively, in the TUDCA and 4-PBA pre-exposed HT + RT treatment arms (Figure 5b,c).

To further link the proteotoxic stress response and HT-induced radiosensitization at the molecular level, we applied the chemical chaperones in both SAS and FaDu spheroids and subsequently analyzed the expression of selected HSR and UPR-related genes by qPCR. As highlighted earlier, 60 min of 42.5 °C HT alone or in combination with RT effectively upregulated some key chaperone (*HSPA1A/B*, *DNAJB1*, *HSPB1*) and ER stress (*sXBP1*, *PPP1R15A*) genes in both spheroid types (Figure S3 *cf.* Figure 4b). TUDCA and 4-PBA pre-treatment alone did not alter the expression of any tested gene (Figure S3). Also, no changes in the HT/HT + RT-induced expression of *HSPA1A/B*, *DNAJB1*, and *CHOP* genes were observed upon TUDCA or 4-PBA pre-treatment (Figure S3). The application of 4-PBA was able to further stimulate the UPR (e.g., *sXBP1* and *PPP1R15A*; Figure 5d and Figure S3). However, 4-PBA pre-administration significantly inhibited the HT/HT + RT-dependent upregulation of *HSPB1* (Hsp27) gene expression (Figure 5d). Hsp27 provides thermotolerance in vivo, supports cell survival under stress conditions, has strong anti-apoptotic activity, and can protect against irradiation [45,46]. Our findings support the hypothesis that the induction of HSR is a protective and pro-survival response in HT + RT treatment outcome.

## 4. Discussion

Multicellular tumor spheroids are a well-established tool in biomedical research and therapy test programs but have less frequently been utilized in systematic HT + RT combination treatment testing. One of the limitations in this context has been the lack of long-term analytical endpoints analogous to the in-vivo read-outs of tumor control probability and tumor control dose 50% reflecting the curative potential of RT in combinatorial treatment settings. We recently established the spheroid control probability (SCP) and spheroid control dose 50% (SCD_50_) as long-term monitoring analytical endpoints for combinatorial radiotherapy testing to better predict the curative potential of new combinatorial treatment strategies before turning to the respective whole animal studies [29,30,31]. The SCP assays is proposed for pre-selecting the most promising combination treatment regimens from a broad range of options whose curative potential cannot be tested in animal models to extend—for ethical and economic reasons. The present study demonstrates the successful adaptation of the SCP assay to evaluate the efficacy of HT in combination with radiation. Our spheroid platform as a whole thus provides a valuable tool to systematically test parameters of interest, which critically affect the therapeutic outcome of HT + RT, such as treatment schedule and appropriate choice of thermal and radiation dose—thereby defining the best treatment settings for further in vivo validation. The SCP approach is both the basis and impetus for an inexpensive, and much more ethical/human assay that has the potential—upon further improvement and validation as highlighted later in this discussion—to more faithfully recapitulate various clinically relevant scenarios in vitro.

We applied two three-dimensional HNSCC spheroid mono-culture models that differ in their intrinsic radioresponse, to re-evaluate the curative potential of HT + RT combination therapy in vitro and to link HT-induced radiosensitization to cellular stress response mechanisms. HNSCC refer to a group of malignant diseases that occur at different anatomical sites, are histomorphologically and genetically heterogeneous, and therapeutically challenging as most patients are diagnosed with large primary cancers or at locoregionally advanced stages [47,48]. The standard-of-care treatment in these cases is multimodal and includes radio(chemo)therapy. However, except for a minor subgroup of patients with tumors related to HPV infections, treatment outcome has remained modest to poor [48]. Resistance to both radiotherapy and the only FDA-approved targeted therapy (anti-EGFR) and subsequent disease relapse is common. Hence, combinatorial treatment options to improve the curative power of radiotherapy are of utmost interest, not only but in particular for patients in the relapsed state. Clinical experience with HT combined with radio(chemo)therapy for recurrent HNSCC is still limited. At present, two registered phase-II clinical trials underway focus on patients with locally recurrent HNSCC: A German study (University Clinic Erlangen) using interstitial HT combined with pulsed-dose-rate brachytherapy (salvage), and a study in Taiwan (Shin Kong Wu Ho-Su Memorial Hospital) with patients treated non-invasively with radio-frequency energy (electromagnetic wave-based) HT concurrent with fractionated external-beam irradiation and chemotherapy (https://www.clinicaltrials.gov; accessed on 19 June 2021). The study designs critically differ. It remains unclear, how such different HT technologies and treatment regimens affect the radiosensitizing potential and responses in HNSCC cells and tissues. Given the wide range of new options for clinical implementation of HT + RT combinatorial treatment setups, ongoing efforts are required allowing the systematic pre-evaluation of various treatment settings in HNSCC models with an in vivo equivalent curative endpoint, and in parallel elucidate those phenomena in HT-induced radiosensitization that are mechanistically still not entirely understood.

Radiosensitization by HT can be attributed to diverse cellular effects and disruption in various cell compartments and protein structures [32,33]. For example, HT induces changes in plasma membrane permeability, alteration in macromolecule synthesis, and intracellular signal transduction. It destroys native chromatin structures, affects DNA double-strand break (DSB) repair, and interferes with DNA damage response (DDR) signaling resulting in increased radiation sensitivity and cancer cell killing [33,49]. In particular, HT inhibits homologous recombination, induces BRCA2 degradation, sensitizes cancer cells to poly(ADP-ribose) polymerase-1 inhibition [50], and affects DNA-PKCs [51]. However, the impact of HT-induced HSR and UPR pathways in the scenario of radiosensitization has still not been sufficiently studied.

Usually, cells integrate multiple stress signals, and the complex interplay of all implicated cellular signaling networks is then decisive in forcing cancer cells into controlled survival or death pathways [52]. HT disrupts thermal protein homeostasis (proteostasis) and causes the accumulation of aberrant proteins in the ER lumen. This process finally results in ER or proteotoxic stress [34,53]. Consequently, eukaryotic cells induce heat shock response (HSR) mechanisms in the presence of thermal stress stimuli by regulating the expression and activation of highly conserved signaling pathways via heat shock proteins (Hsps/chaperones) [34,54]. Hsps maintain proteostasis by enabling de novo protein folding in the overloaded intracellular environment and by targeting aberrant proteins for degradation [55,56]. Heat shock factor 1 (HSF1) is an essential transcription factor, which coordinates a transcriptional program consisting of Hsp genes in order to increase protein-folding capacity, rebalance proteostasis and support cellular function [57,58]. Moreover, accumulation of aberrant proteins triggers the unfolded protein response (UPR) signaling pathways to restore normal proteostasis, supporting cell survival and adaptation [35]. Under massive HSR and unresolvable ER stress conditions, the UPR integrates cell-fate decision signals and activates a particular cell death program [52,59]. On the other hand, systemic HT can lead to development of thermotolerance, as cancer cells lose their susceptibility to heat due to the persistent overexpression of Hsps and triggered UPR [60].

A global overview of the transcriptional changes in the two spheroid types upon HT and HT + RT treatments indicates modulations in fundamental stress response mechanisms. These modifications are mainly triggered by the exposure to HT and affect the protein folding machinery and quality control besides survival and cell death signaling pathways. All modulations are integral to HSR and proteotoxic stress responses to support the cells’ survival, recovery, and adaptation to the stressful conditions [61,62]. Accordingly, HT + RT induced significant modulations in primary cellular stress-response signaling in both spheroid types, e.g., in PKB/Akt, MAPK, and NF-κB pathways. As an example, ERK1/2, a well-characterized MAPK activated in response to growth stimuli, is unaltered by HT + RT, while JNK and p38 are significantly affected. Both are MAPK stress kinases known to be activated in response to various internal and external stresses such as DNA damage, heat shock, inflammatory cytokines, changes in osmolarity or metabolism, ischemia, UV irradiation, oxidative stress, etc. [63]. In our study, the strong activation of JNK and p38 stress kinases in both HNSCC spheroid models upon HT mono- and HT + RT combination treatments resulted in a powerful induction of c-Jun. The latter is considered as global transcription factor and has diverse functions in apoptosis regulation, protecting DNA repair and cell cycle progression depending on the biological context and cell type [42,43].

Our results further indicate that HT’s main target is the cellular proteome, leading to the imbalance in proteostasis and activation of HSR and UPR signaling. The activation status of HSR, mediated by HSF1, selected chaperons, and UPR signaling, was markedly upregulated in both FaDu and SAS spheroids after HT and RT. The findings agree with previous studies that examined genetic networks responsive to mild HT in different 2-D cancer cell cultures [64,65]. We propose that the HT-induced imbalance in cellular proteostasis, its severity, and the cells’ capacity to activate appropriate adaptive and survival signaling programs affect the radiosensitization efficiency (as observed in the SCP assay). HT at higher (44–46 °C) temperatures induced more severe and prolonged proteome damages and UPR-signaling while causing a more substantial radiosensitization.

In principle, HT induced strong radiosensitization in both HNSCC models. However, the effect was clearly more pronounced in the intrinsically more radioresistant, thermosensitive HNSCC spheroid model SAS. Whole-genome profiling revealed numerous differences in the baseline gene expression patterns of SAS and FaDu spheroids (Figure S4a,b), which might partially be attributed to the phenotypic and functional heterogeneity of HNSCC and the different tissue of origin [47,48]. Paradoxically, we found significantly higher expression of some key chaperones (e.g., *HSPA2*, *HSPB8*) and a lower intrinsic expression of other Hsp genes in the more thermoresistant FaDu. How this differential baseline expression of chaperone proteins may underline the subsequent difference in heat sensitivity needs to be further determined. However, our data imply that FaDu spheroids can more efficiently activate protective heat defense mechanisms (HSR and UPR) than SAS spheroids. Furthermore, FaDu spheroid cells may already be primed to counteract the heat stress more efficiently. The incidential finding that *HSPB1* gene expression is upregulated exclusively in FaDu but not SAS cells in the 3-D environment compared to monolayer culture supports this hypothesis (Figure S4c). Hence, FaDu cells in the 3-D setting may better adapt and survive via active stress response signaling (e.g., MAPK pathways), thereby reducing HT’s radiosensitizing potential. By contrast, the less efficient induction of the chaperone machinery and UPR signaling in SAS spheroid cells results in a more severe damage of the cellular proteome and putative initiation of cell death programs [66]. Interestingly, the cleaved form of PARP protein, a well-known marker of apoptosis, started to accumulate exclusively in SAS spheroids after 24 h of high-dose RT or HT + RT treatments. Although some mechanistical details remain unsolved, our data demonstrate an integral and complex role of proteotoxic stress response in HT-induced radiosensitization.

To functionally strengthen this interpretation, HNSCC spheroids were exposed to two well-known chemical chaperones with different abilities to alleviate proteotoxic stress before and during HT with and without radiation. TUDCA, a secondary bile acid that mitigates heat-induced protein aggregation [67], slightly increased the proportion of controlled SAS spheroids, but no link to alterations in gene expression was indicated in our data set (derived from measurements 30 min after treatment). 4-PBA, which suppresses UPR and promotes ER stress-induced cell death [67], intensified the heat-induced radiosensitization even more. Notably, 4-PBA can also act as a histone deacetylase inhibitor and interfere with post-translational modification of histones, thereby suppressing general stress response signaling [68]. On the molecular level, 4-PBA pre-treatment altered the HT/HT + RT-induced gene expression of *sXBP1* (increase) and *HSPB1* (decrease). We will therefore illuminate the putative role of these molecules in the context of HT-induced radiosensitization.

Activation of the IRE1α-XBP1 pathway usually constitutes a pro-survival response during UPR [52]. Recently published data from our group demonstrated that SAS cells are in general more sensitive to the XBP1 splicing reaction than FaDu cells, implying that SAS cells require a lower stress threshold [38]. This also holds true for the reaction to HT, i.e., *XBP1*-splicing was more pronounced at lower thermal doses in the SAS than the FaDu spheroid model. In the previous study, we found that via knockdown of the ER stress sensor IRE1, the IRE1α-XBP1 pathway does not play a role in metabolic stress-induced radiosensitization in SAS cells [38]. We, therefore, hypothesize that the upregulation of XPB1 splicing upon HT/HT + RT, further enhanced by 4-PBA, also just reflects the cells’ attempt for survival but supposedly fails. In contrast to that, 4-PBA significantly suppressed the HT/HT + RT-induced expression of Hsp27 in the HNSCC spheroids. This *HSPB1* (Hsp27) down-regulation is expected to interfere with its known protective function in cells. In this context, a previous study demonstrated a protective role of *HSPB1* against radiation-induced apoptosis and radiosensitization by increasing glutathione levels for ROS detoxification and delaying mitochondrial collapse and caspase activation [46]. Whether this is the only functional link remains to be elucidated. However, altogether our data suggest that HSR and proteotoxic stress-induced signals can interfere with HT-induced radiosensitization. Consequently, their co-targeting might improve RT outcome as indicated for various tumor cells, including HNSCC [62,66,69,70].

The theranostic value of selective chaperones in malignancies and their dualistic role in tumor cell protection and destruction is currently under extensive investigation [36,69]. For example, last-generation Hsp90 inhibitors are now in clinical trials and show promising results in combination with RT for certain solid cancers [71]. Hsp27 inhibitors were also developed and characterized, although Hsp27 as an ATP-independent chaperone appears difficult for small molecule targeting [72,73]. The most notable example is the second-generation antisense oligonucleotide drug apatorsen (OGX-427), which proved therapeutic activity in various animal cancer models and clinical trials on castration-resistant prostate cancer and other advanced malignancies [74,75]. The new inhibitor of Hsp27 phospho-activation ivermectin has been described more recently and shows promising biochemical and functional mechanisms of action in tumor cells in vitro and in vivo [76].

Together with these developments, our observations in the spheroid assay call for and justify follow-up studies to combine Hsp27 inhibitors with HT + RT in HNSCC 3-D models in vitro and, even more importantly, in vivo. Best case, these extended studies shall also consider the immunotherapeutic potential of HT, which may further enhance tumor radioresponse. The HT-induced immunogenic reaction is affected by Hsps and triggered via stress and pro-inflammation NF-κB signal pathways in immune cell populations [77,78]. This aspect could not yet be covered by the current experimental design using the intriguing long-term analytical endpoints of SCP and SCD_50_. The development of extended SCP assays to include stromal and immune cells is one focus of our ongoing work, as is the application of more clinically relevant fractionation regimes for combinatorial treatment testing.

## 5. Conclusions

The long-term analytical endpoints of spheroid control probability and spheroid control dose 50% are key elements of our 3-D assay platform that critically improve the informative value of in vitro HT + RT testing even beyond spheroid volume growth. In this light, spheroids, despite the recognized limitations, present a powerful preclinical tool to compare various treatment regimens, e.g., with respect to heating technology, duration, and sequence of HT/RT treatment or combination with chemotherapeutics and targeted therapies, as a prerequisite to select the most promising HT + RT settings before turning into whole animal studies.

The current study provides new insights into molecular mechanisms interfering with HT-mediated radiosensitization. Our observations indicate that massive HSR and induction of proteotoxic stress signals can, to some extent, protect cells from HT-induced radio-sensitization. Hsps, e.g., Hsp27, appear to be critical in this scenario. We, therefore, propose to evaluate the inhibition of particular Hsps as a multi-targeting strategy to overcome this phenomenon for improving the outcome of combinatorial HT + RT. Since the intrinsic expression of stress response genes may differentially change from the 2-D to 3-D environment, 3-D cell assays should be integral to the identification and primary screening of candidate therapeutics directed against putative druggable targets for combination with HT and RT. Preclinical in vivo testing and the validation of the respective Hsps as reliable predictive biomarkers shall then further guide the future development of such an approach for HNSCC treatment.

## Figures and Tables

**Table 1:** SCD_50_ and TER values for FaDu and SAS spheroids pre-exposed to different HT doses directly before irradiation. The data were extracted from the SCP curve fittings using function (1), shown in Figure 2c. All treatment-related changes in SCD_50_ are significant at a level of *p* << 0.001 (see Materials and Methods for details on statistics using the pooled data sets from N = 2 independent experiments and Table S4 for interexperimental reproducibility of the TER values).

Spheroid Type	Readout	RT Only	HT + RT			
			42.5 °C 30 min	42.5 °C 60 min	44.5 °C 30 min	46.5 °C 30 min
FaDu	SCD_50_ (Gy)	11.1	8.7	7.3	7.5	3.2
	95% CI (Gy)	10.8–11.3	8.4–9.1	7.1–7.6	7.3–7.8	3.0–3.6
	TER		1.3	1.5	1.5	3.4
	95% CI		1.2–1.3	1.4–1.6	1.4–1.5	3.1–3.8
SAS	SCD_50_ (Gy)	16.5	12.4	10.2	8.0	3.5
	95% CI (Gy)	16.2–16.8	12.2–12.7	9.9–10.5	7.7–8.3	3.3–3.9
	TER		1.3	1.6	2.1	4.6
	95% CI		1.3–1.4	1.6–1.7	2.0–2.1	4.3–5.0

## Data Availability

The accession number for the RNA sequencing data reported in this paper is NCBI GEO: GSE150922.

## Supplementary Materials

The following are available online at https://www.mdpi.com/article/10.3390/cancers13133168/s1, Figure S1. Methodological prerequisites, Figure S2. HT induces more severe and prolonged proteome damage and UPR signaling in HNSCC spheroids at higher temperature, Figure S3. The pre-exposure to chemical chaperones only modifies the expression of some selected HT-induced HSR and UPR genes in HNSCC spheroids, Figure S4. HNSCC spheroid models have substantial differences in the baseline genomic expression profiles; HSPB1 gene expression massively changes from 2-D to 3-D culturing only in the FaDu model, Table S1. Primers used for the detection and quantification of HSR genes by RT- and qPCR analysis, Table S2. List of antibodies used in the study, Table S3. Relative volume growth delay induced in FaDu and SAS spheroids by different doses of HT, Table S4. The TER values calculated individually from two SCP experiments performed for each spheroid type show small interexperimental variability, Table S5. List of significantly (2-fold change or more, adjusted *p* ≤ 0.05) unique and common regulated genes under RT, HT and HT + RT treatments in FaDu and SAS spheroids, Table S6. List of significantly (2-fold change or more, adjusted *p* ≤ 0.05) over-represented GO terms in FaDu and SAS spheroids 0.5 h after HT + RT compared to untreated control spheroids using the GO enrichment analysis.
