# Supplementary material for: Efficient Heat Shock Response Affects Hyperthermia-Induced Radiosensitization in a Tumor Spheroid Control Probability Assay
Source: Cancers (Basel). 2021 Jun 25;13(13):3168. doi: 10.3390/cancers13133168 (PMC8269038; doi:10.3390/cancers13133168)
Supplement: Supplementary file 1 [file cancers-13-03168-s001.zip › Figures S1-S4.pdf]

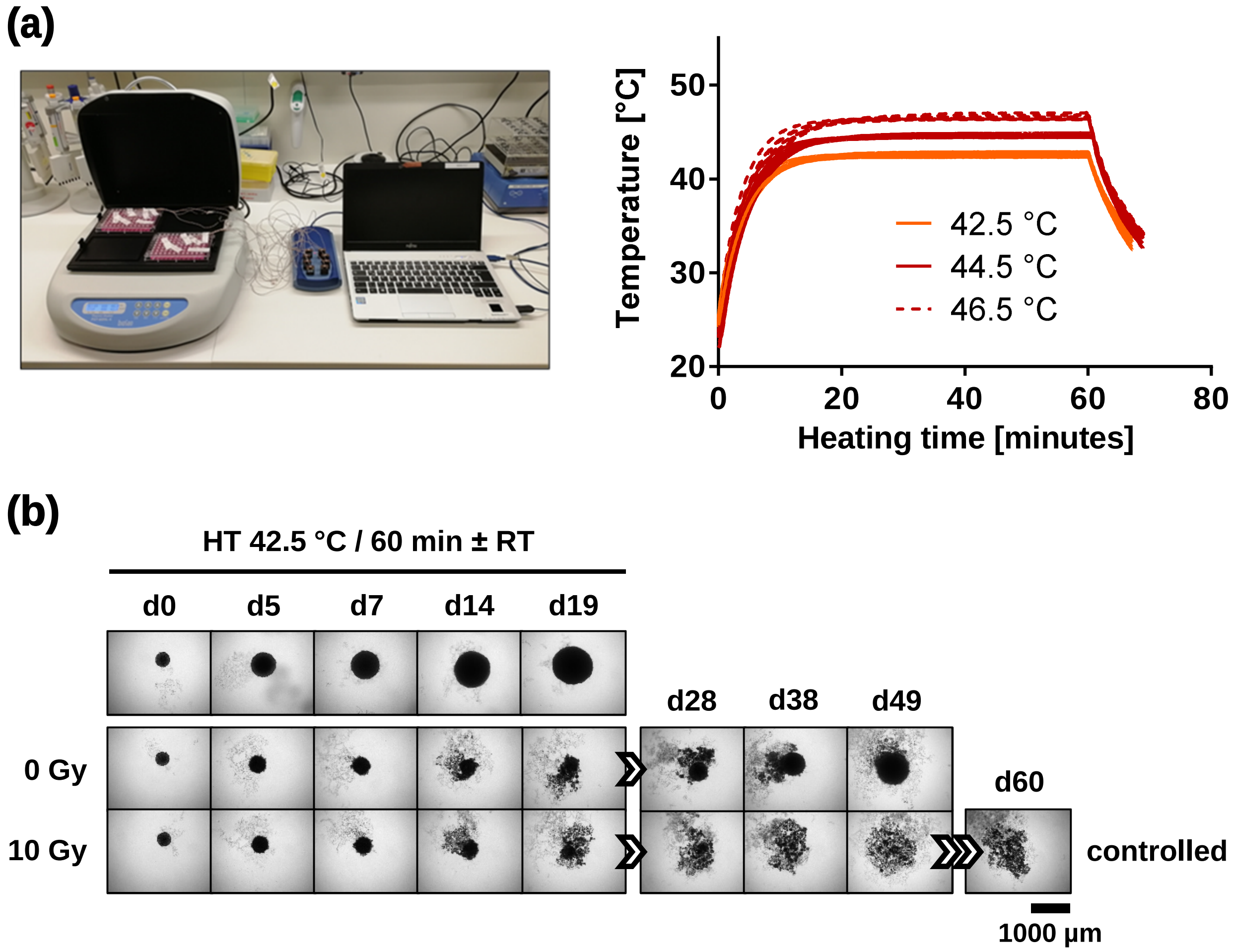

Figure S1. Methodological prerequisites: (a) PST-60HL-4 Plate Thermo-Shaker (BioSan, Latvia) for exposure of spheroids to HT. The left panel shows the setup for the recording of the heating profiles in the wells of two agarose-coated 96-well plates; representative heating profiles recorded for different temperature settings with the TC-08 8-channel thermocouple data logger (Pico technology, UK) are documented in the right panel. **(b)** Long-term readout in the spheroid control probability assay: Image series of a representative SAS spheroid after HT mono-treatment (42.5 °C / 60 min) showing continuous growth (upper panel) and of two spheroids (lower panel) that were both irradiated with 10 Gy single dose X-ray after HT but show variable response, i.e., one spheroid recovers and regrows after some delay, while the other one remains growth-controlled ("cured") over the monitoring time of 60 days post-treatment. Multiple spheroids were monitored accordingly in each treatment arm to assess proportion of controlled spheroids (=SCP).

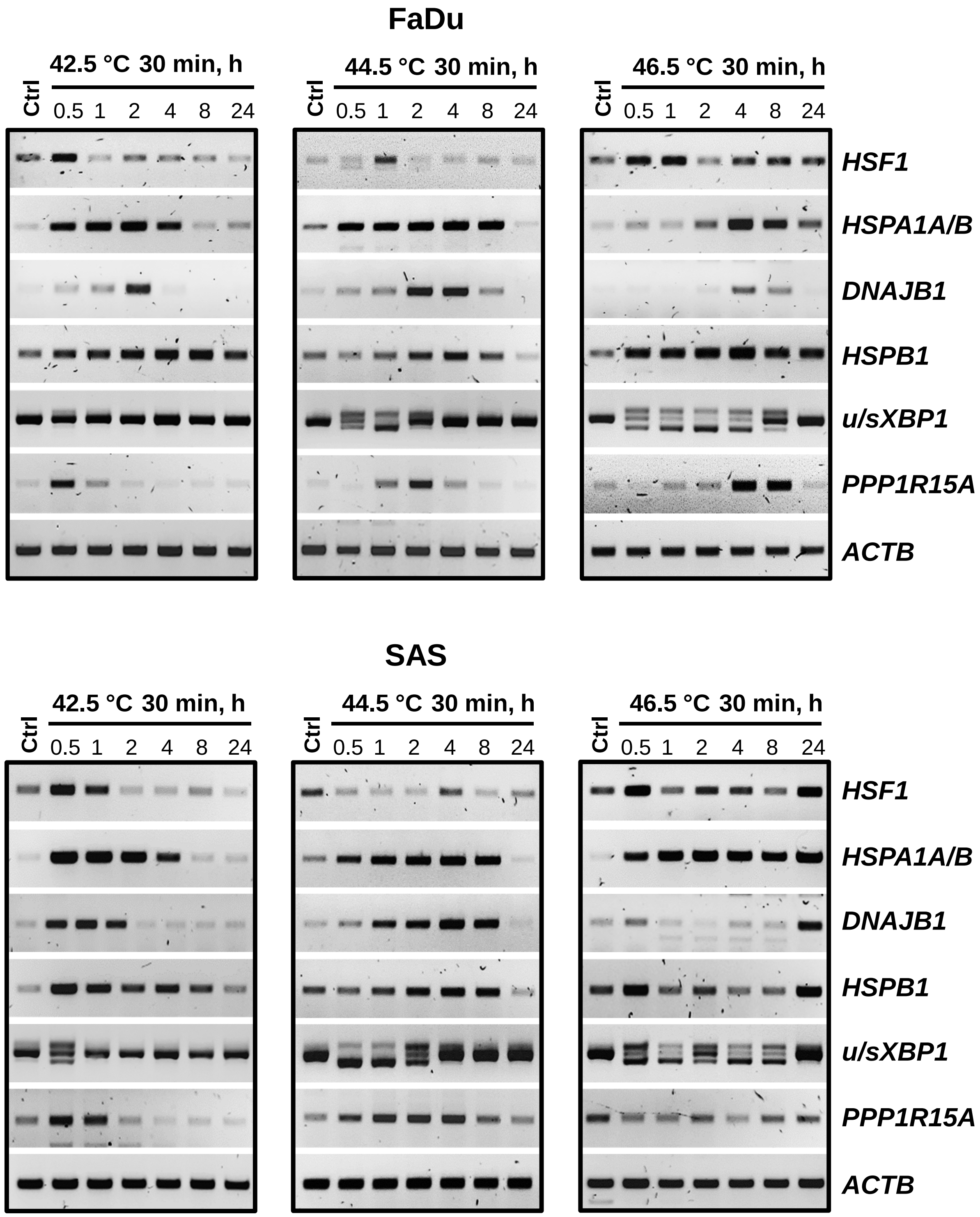

Figure S2. HT induces more severe and prolonged proteome damage and UPR signaling in HNSCC spheroids at higher temperature: Representative data sets of HSR genes in **(a)** FaDu and **(b)** SAS spheroids analyzed by RT-PCR. FaDu and SAS spheroids were treated with different settings of HT (42.5 °C, 44.5 °C, and 46.5 °C / 30 min) and analyzed at 0.5-24 h post-treatment. *ACTB* mRNA level was routinely determined as reference; ctrl – untreated control spheroids.

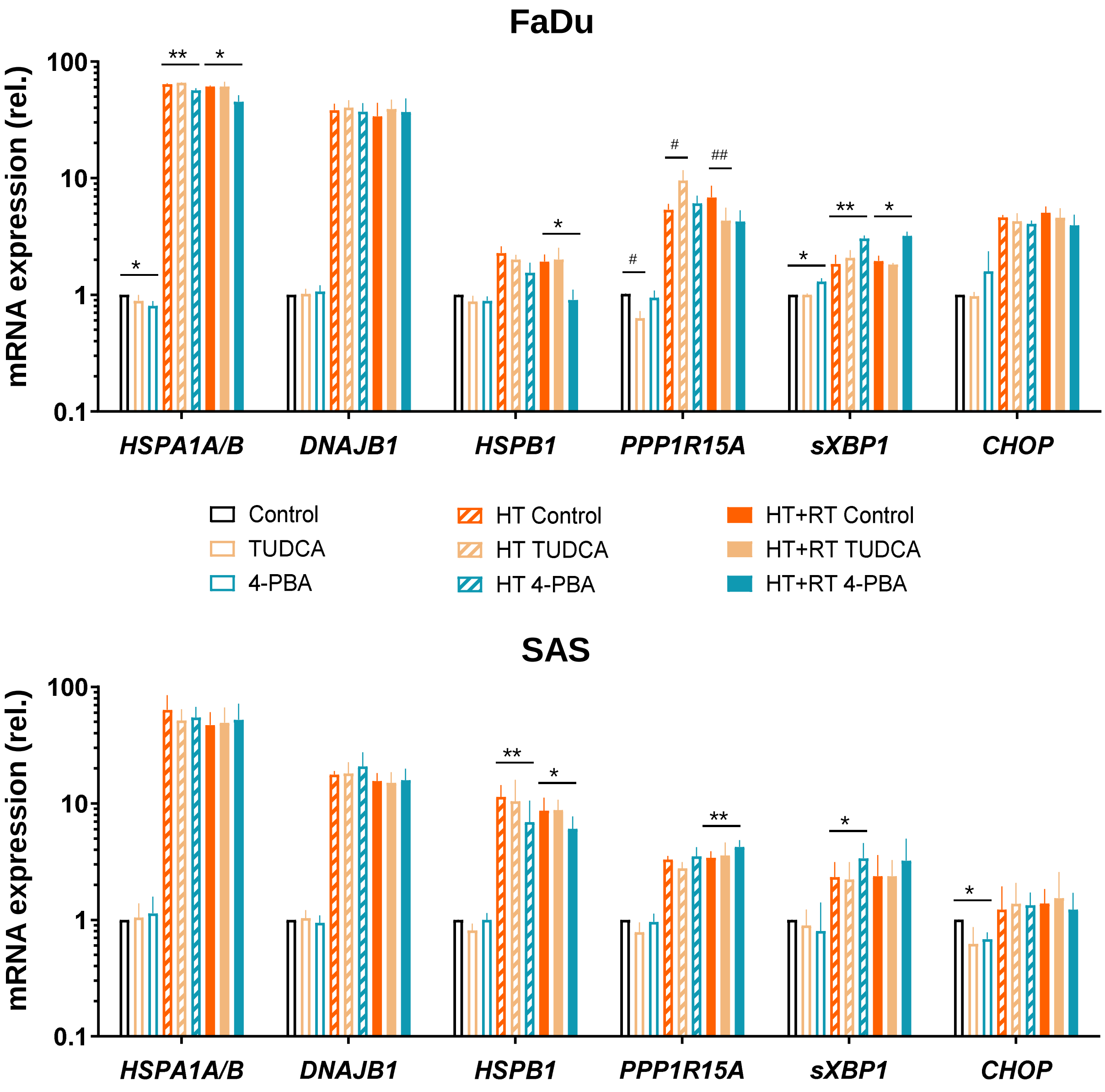

Figure S3. The pre-exposure to chemical chaperones only modifies the expression of some selected HT-induced HSR and UPR genes in HNSCC spheroids: q-PCR analysis of several HSR and UPR genes identified as upregulated in HNSCC spheroids upon HT mono- and/or HT+RT combination treatment FaDu (upper panel) and SAS spheroids (lower panel) were exposed to the chemical chaperones TUDCA (0.4 mM) or 4-PBA (5 mM) before and during HT and HT+RT, respectively (42.5 °C / 60 min; 7 Gy for .FaDu and 10 Gy for SAS spheroids) . Data normalized to *ACTB* gene expression levels from N=3 experiments are shown on a logarithmic scale as means (+SD); #p<0.05, ##p<0.01 - TUDCA-treated versus untreated; *p<0.05, **p<0.01 - 4-PBA-treated versus untreated.

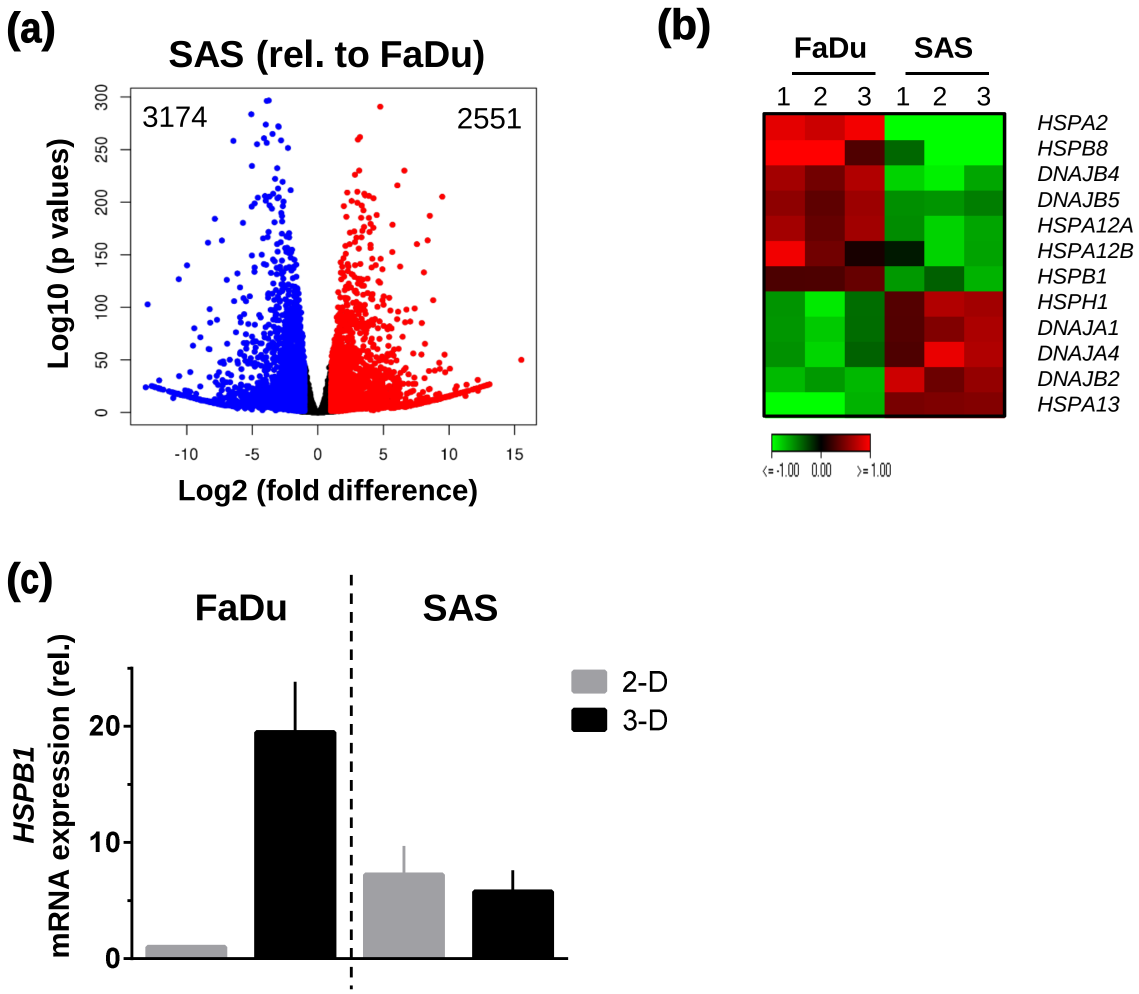

Figure S4. HNSCC spheroid models have substantial differences in the baseline genomic expression profiles; *HSPB1* gene expression massively changes from 2-D to 3-D culturing only in the FaDu model (a) Volcano plot showing the distribution of differentially expressed genes between control FaDu and SAS spheroids profiled by RNA-Seq analysis in triplicate. The x-axis represents log2 (SAS/FaDu) values for each gene, and the y-axis shows statistical significance. Red dots indicate highly expressed genes and blue dots are genes with lower expression in SAS versus FaDu spheroids (adjusted p<0.05); black dots are genes with less than 2-fold difference. **(b)** Heat map of differentially expressed chaperone genes in untreated FaDu and SAS spheroids profiled by RNA-Seq analysis in triplicate. Genes were selected by DESeq2 with at least 2-fold difference in expression (adjusted p<0.05). Data shown are log2 values color-coded from green (lower expression) to red (higher expression). **(c)** q-PCR analysis of intrinsic *HSPB1* gene expression in FaDu and SAS cells in exponentially growing monolayer cultures and spheroids with a mean diameter of ~400 µm at day 4 after inoculation; *ACTB* mRNA levels served as reference, and the expression data were further related to the averaged *HSPB1* expression in FaDu monolayer cells. The graph shows mean relative mRNA expression values from N≥3 independent samples (n=2 technical replicates/sample) for each cell line and culture condition.
