# Supplementary material for: Efficient Heat Shock Response Affects Hyperthermia-Induced Radiosensitization in a Tumor Spheroid Control Probability Assay
Source: Cancers (Basel). 2021 Jun 25;13(13):3168. doi: 10.3390/cancers13133168 (PMC8269038; doi:10.3390/cancers13133168)
Supplement: Supplementary file 1 [file cancers-13-03168-s001.zip › Tables S1-S4.pdf]

Table S1. Primers used for the detection and quantification of HSR genes by RT- and qPCR analysis.

Target gene	Primer sequence	Product size
ACTB	Forward - CACCCTGAAGTACCCCATCG<br>Reverse - GCTGGGGTGTTGAAGGTCTC	199 bp
DNAJB1	Forward - GTCATTTATCCTGCCAGGATCAG<br>Reverse - GTCCCCACGTTTCTCGGGTG	185 bp
HSF1	Forward - CACAACAACATGGCCAGCTTCG<br>Reverse - CCTGGCGGATCTTTATGTCTTC	217 bp
GADD34/PPP1R15A	Forward - GAGACAGAGGAAGAGGAAGCT<br>Reverse - GGAAATGGACAGTGACCTTCTC	235 bp
HSPA1A/B	Forward - CCAGGTGATCAACGACGGAGAC<br>Reverse - CGATCACACCCGCATCCTTGG	218 bp
HSPB1	Forward - CGCCATCGAGAGCCCCGCAG<br>Reverse - CCTGCCGCTCCTCGTGCTTGC	203 bp
XBP1***	Forward - CCTTGTAGTTGAGAACCAGG<br>Reverse - GGGGCTTGGTATATATGTGG	442 bp<br>416 bp

*Primers for unspliced and spliced forms of the f *XBP1* gene were according to Yoshida *et al*., 2001 [44]

Table S2. List of antibodies used in the study.

Antibody	Source	Cat. Number
Rabbit monoclonal antibody anti-ATF4	Cell Signaling Technology	# 11815S
Mouse monoclonal antibody anti-AKT	Cell Signaling Technology	# 2920S
Rabbit monoclonal antibody anti-Phospho-AKT (Ser475)	Cell Signaling Technology	# 4060S
Rabbit monoclonal antibody anti-Phospho-p44/ 42 MAPK (Erk1/2) (Thr202/Tyr204)	Cell Signaling Technology	# 4370S
Rabbit polyclonal antibody anti-p44/42 MAPK (Erk1/2)	Cell Signaling Technology	# 9102S
Rabbit monoclonal antibody anti-Phospho-eIF2alpha (Ser51)	Cell Signaling Technology	# 3597S
Rabbit monoclonal antibody anti-cleaved PARP (Asp214)	Cell Signaling Technology	#9541S
Rabbit polyclonal antibody anti-LC3B	Cell Signaling Technology	# 4108S
Rabbit monoclonal antibody anti-Phospho-SAPK/JNK (Thr183/Tyr185)	Cell Signaling Technology	#4668S
Rabbit polyclonal antibody anti-SAPK/JNK	Cell Signaling Technology	#9252S
Rabbit monoclonal antibody anti-Phospho-p38 MAPK (Thr180/Tyr182)	Cell Signaling Technology	#4511S
Rabbit monoclonal antibody anti-p38 MAPK	Cell Signaling Technology	#8690S
Rabbit monoclonal antibody anti-Phospho-MAPKAPK-2 (Thr334)	Cell Signaling Technology	#3007S
Rabbit monoclonal antibody anti-Phospho-c-Jun (Ser73)	Cell Signaling Technology	#3270S
Mouse monoclonal antibody anti-HSP70	Cell Signaling Technology	#46477S
Rabbit monoclonal antibody anti-HSP40	Cell Signaling Technology	#4871S
Rabbit monoclonal antibody anti-Phospho-HSP27 (Ser82)	Cell Signaling Technology	#9709S
Mouse monoclonal antibody anti-HSP27	Cell Signaling Technology	#2402S
Rabbit polyclonal antibody anti-HSF1	Cell Signaling Technology	#4356S
Rabbit monoclonal antibody anti-Phospho-HSF1 (Ser326)	Abcam	ab76076
Rabbit polyclonal antibody anti-Phospho-IRE1 alpha (Ser724)	Novus	NB100-2323
Rabbit monoclonal antibody anti-Phospho-ATM (Ser1981)	Cell Signaling Technology	#5883S
Rabbit polyclonal antibody anti-Phospho-ATR (Ser428)	Cell Signaling Technology	#2853S
Rabbit monoclonal antibody anti-Phospho-Histone H2A.X (Ser139)	Cell Signaling Technology	#9718S
Rabbit monoclonal antibody anti-Atg5	Cell Signaling Technology	#9980S
Rabbit monoclonal antibody anti-GAPDH	Santa Cruz Biotechnology	sc-47724

Table S3. Relative volume growth delay induced in FaDu and SAS spheroids by different doses of HT. Relative volume growth delay was calculated by dividing the individual time periods required by each treated spheroid to reach $5xV_0$ (volume before treatment) by the average time of control spheroids (37 °C) to get to the same endpoint. Values are documented as means ± SD.

Temperature [°C]	Treatment time [min]	Growth delay (rel.)	
		FaDu	SAS
42.5 °C	30	1.0 ± 0.1	1.0 ± 0.1
42.5 °C	60	1.0 ± 0.1	1.2 ± 0.1
44.5 °C	30	1.0 ± 0.1	1.5 ± 0.1
46.5 °C	30	1.6 ± 0.4	3.3 ± 0.4

Table S4. The TER values calculated individually from two SCP experiments performed for each spheroid type show small interexperimental variability. Spheroid dose-response curve fitting was performed according to Figure 2c as described in Materials and Methods but for each experiment individually (n≥26 spheroids were monitored per radiation dose group in all HT treatment arms). TERs with 95% confidence interval (CI) were determined via the SCD_{50} values derived from the SCP curves.

Spheroid type		Readout	HT+RT			
			42.5 °C 30 min	42.5 °C 60 min	44.5 °C 30 min	46.5 °C 30 min
FaDu	Experiment 1	TER	1.2	1.6	1.5	3.5
		95% CI	1.2-1.3	1.5-1.7	1.4-1.7	3.0-4.1
	Experiment 2	TER	1.2	1.5	1.4	2.9
		95% CI	1.2-1.3	1.4-1.6	1.3-1.5	2.7-3.1
SAS	Experiment 1	TER	1.3	1.7	1.9	4.5
		95% CI	1.2-1.4	1.6-1.7	1.8-2.0	4.0-5.0
	Experiment 2	TER	1.3	1.6	2.1	4.7
		95% CI	1.3-1.4	1.5-1.7	2.0-2.2	4.3-5.1
